# 12-Month trajectories of physical and mental symptom scores after COVID-19 hospitalization and their role in predicting “very long” COVID

**DOI:** 10.3389/fresc.2025.1568291

**Published:** 2025-05-21

**Authors:** Oleksii Honchar, Tetiana Ashcheulova, Alla Bobeiko, Viktor Blazhko, Eduard Khodosh, Nataliia Matiash, Vladyslav Syrota

**Affiliations:** ^1^ Department of Propedeutics of Internal Medicine, Nursing and Bioethics, Kharkiv National Medical University, Kharkiv, Ukraine; ^2^ Department of Pulmonology, MNE “Clinical City Hospital No.13” of Kharkiv City Council, Kharkiv, Ukraine

**Keywords:** long COVID, post-COVID syndrome, survey-based assessment, machine learning, recovery trajectories, rehabilitation

## Abstract

**Background:**

Long COVID syndrome (LCS) represents a significant global health challenge due to its wide-ranging physical and cognitive symptoms that persist beyond 12 months in a substantial proportion of individuals recovering from SARS-CoV-2 infection. Developing tools for predicting long-term LCS persistence can improve patient management and resource allocation.

**Objective:**

To evaluate the natural dynamics of symptoms over 12 months following hospitalization for COVID-19 and to establish the utility of survey-based symptoms assessment for predicting LCS at one year.

**Methods:**

This prospective observational study included 166 hospitalized COVID-19 survivors who were evaluated pre-discharge and followed up at 1, 3, and 12 months. Assessments included surveys including physical and mental symptom scales (e.g., EFTER-COVID, SBQ-LC, PCFS, MRC Dyspnea, CAT, CCQ, and HADS) and machine learning modeling to predict LCS persistence at 12 months.

**Results:**

LCS symptoms were reported by 76% of patients at three months and 43% at 12 months. Physical symptom scores, particularly EFTER-COVID and PCFS, consistently differentiated LCS and LCS-free cohorts. CAT outperformed other respiratory scales in its discriminatory ability, while HADS subscales showed limited predictive value. Younger patients (<40 years) demonstrated faster recovery, whereas older patients (>60 years) exhibited persistent symptoms across respiratory and cognitive domains. A machine learning model combining EFTER-COVID, SBQ-LC, CAT, and MRC Dyspnea scores achieved 91% predictive accuracy for LCS persistence at 12 months.

**Conclusion:**

Comprehensive survey-based symptoms assessment at three months post-discharge provides a practical and cost-effective tool for prediction of the long COVID persistence at 12 months, supporting targeted rehabilitation strategies.

## 1 Background

A lot of attention has been drawn recently to the problem of the long-term persistence of symptoms following the acute phase of SARS-CoV-2 infection. Despite the end of COVID-19-related global health emergency that the WHO announced in May 2023 (1), healthcare systems worldwide continue to deal with an emerging non-transmissible pandemic presented by the long COVID syndrome (LCS). Its prevalence among the SARS-CoV-2 infection survivors differs depending on the definitions used, population characteristics, and acute phase severity (with the need for hospitalization being the simplest and most reliable marker of the latter), which also complicates assessment of the LCS duration. At the same time, both a recent symptom-based meta-analysis (2) and a large cohort study that utilized a self-reported post COVID recovery assessment (3) came up with similar findings: roughly half of the participants continued to experience at least one new symptom or sign of incomplete recovery beyond 12 months after acute phase; the term “very long COVID” has been recently proposed by Ranucci et al. to describe this scenario (4).

Considering the scope of the most typical long-lasting post-acute COVID-19 sequelae that include dyspnea, fatigue, chest and joint pain, neuropsychiatric disorders, and cognitive dysfunction, LCS frequently presents as a functionally limiting condition that does not only affect quality of life but may also cause varying degree of disability (5, 6). In this setting, the development of prognostic tools predicting both the occurrence and long-lasting persistence of LCS might prove highly beneficial for optimizing the allocation of resources directed to rehabilitaion of post-acute COVID-19 patients and forecasting the need for support programs; currently, there is a lack of such tools.

We have previously presented the proceedings of the machine learning approach to the early prediction of negative post-discharge outcomes (defined as poor physical recovery at 1 month and self-reported incomplete recovery at 3 and 12 months) largely based on pre-discharge parameters (7–9). The current analysis aimed to study the natural dynamics and additive prognostic value of the selection of physical and mental symptoms scales' scores that present cheap and easily available parameters with a hypothetic capacity to further improve the accuracy of predicting the development of the “very long” COVID syndrome.

### 1.1 Objective

To evaluate the natural dynamics of symptoms over 12 months following hospitalization for COVID-19 and to establish the utility of survey-based symptoms assessment for predicting LCS at one year.

## 2 Materials and methods

### 2.1 Study design and population

The current prospective observational study was conducted at a single specialized COVID-19 care center in Kharkiv, Ukraine, between January and November 2021 (at the time of recruiting, the area served was inhabited by 2.4 mln people). During this period, eligible patients who were hospitalized with the preliminary diagnosis of pneumonia or COVID-19 were invited to participate in the study. Eligibility criteria included confirmed SARS-CoV-2 etiology, for which polymerase chain reaction test was used, and the age of ≥18 years (refer to the Supplementary Material for exclusion criteria).

**Figure 1:**
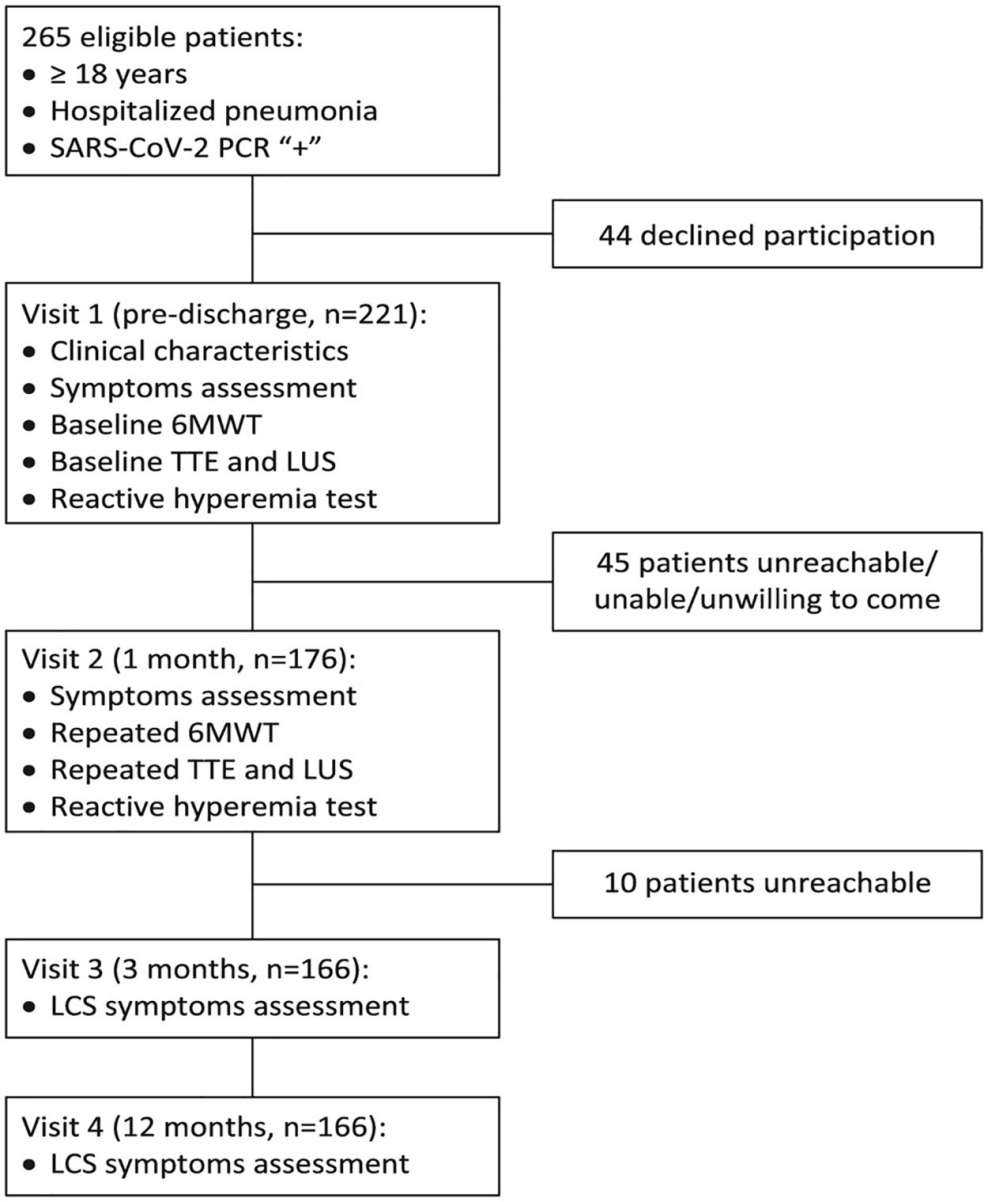
Study flowchart.

Out of the total of 265 consecutive eligible patients that were identified, 44 have declined the invitation to participate, and 221 patients were enrolled. The complete baseline and follow-up data was available on 166 participants who made up the final study cohort (see Figure 1 for the detailed study flow chart).

### 2.2 Clinical data collection

The first visit was performed at the end of the hospitalization period, after clinical stabilization of the patients and reaching the criteria of epidemic safety that included normal (>93%) oxygen saturation (SpO2) on room air, absence of symptoms and signs of acute respiratory disease along with normalization of body temperature for ≥3 days, counting from the 10th day after onset of acute COVID-19, as recommended by the WHO (10). This visit was used to obtain data on baseline demographic characteristics, treatment used, laboratory and instrumental findings, and anthropometrical parameters from the medical records, and to collect medical history data through the interview. Follow-up visits were performed at 1 month in person, and at 3 and 12 months through telephone, text messengers, or e-mail. During each visit, participants were asked to complete a set of questionnaires on symptoms that included CAT and CCQ to assess respiratory symptoms, EFTER-COVID study subscale on physical symptoms, HADS to assess anxiety and depression levels, and SBQ-LC questionnaire subscale on Memory, Thinking and Communication (pre-disease scores on EFTER-COVID and SBQ-LC subscales were obtained retrospectively during the follow-up visit at 1 month) (11–15). General physical functional status was assessed using the MRC Dyspnea scale and Post COVID functional scale (PCFS) (16, 17). LCS was defined as a self-reported incomplete recovery of health status or persistence of new symptoms compared to pre-COVID-19 state which was assessed at 1, 3, and 12 months post-discharge.

### 2.3 Statistical analysis

The data analysis was performed using StatSoft STATISTICA 12 software suite. Shapiro–Wilk test was used to assess the conformity of generated data to normal distribution. For continuous parameters, normally distributed variables are expressed as mean ± standard deviation (SD), and skewed ones as median [interquartile range]. Categorical variables are expressed as counts (percentages). Inter-group comparisons were performed using independent samples t-test for normal and Mann–Whitney U-test for non-normal distributions. Longitudinal comparisons were performed using paired samples t-test and Wilcoxon signed-rank test, respectively. Categorical and binary variables were compared using the Chi-Square test. Statistical significance was acknowledged in *p* < 0.05.

The predictive value of the studied parameters was primarily gauged by their marginal effects in logistic regression analysis. Subsequently, significant predictors of LCS (defined as Wald *p*-value < 0.05) were used as inputs in the development of multiple binary classification models using different machine learning (ML) methods, including support vector machines (SVM), naive Bayes, K-nearest neighbors, random forest, boosted trees, and simple artificial neural networks (SANN) utilizing an automated neural architecture search strategy based on random selection of the number of hidden units and MLP activation functions (Identity, Logistic, Tanh, Exponential) with subsequent retaining of the networks with the highest predictive accuracy. The process of selecting subsets for training, testing, and validation was performed via random sampling, preserving the proportions of 70:15:15 within the study cohort. A total of 500 binary classification models were developed for each configuration of input variables while developing SANN-based models, and a maximum of 500 trees was set while employing random forest and boosted trees methods (this quantity was determined empirically as a balance between computational resource allocation and the model reproducibility, with fewer models count resulting in fluctuating predictive accuracy). Missing data entries were imputed with mean values to preserve sample size and models stability, given a limited number of cases. The predictive accuracy of the resultant models was evaluated based on the percentage of correctly classified cases within the test and validation subsets, sensitivity, specificity, positive and negative predictive values. For the optimally performing predictive models obtained by each method, k-fold cross-validation was used with a minimal *k* = 10. For the final classification model, receiver-operator curve (ROC) analysis has been performed. *Post-hoc* evaluation of sample size adequacy was performed that was based on the assessment of model accuracy and dataset effect size utilizing Cohen's d statistic (18).

## 3 Results

### 3.1 Baseline characteristics

**Figure 2:**
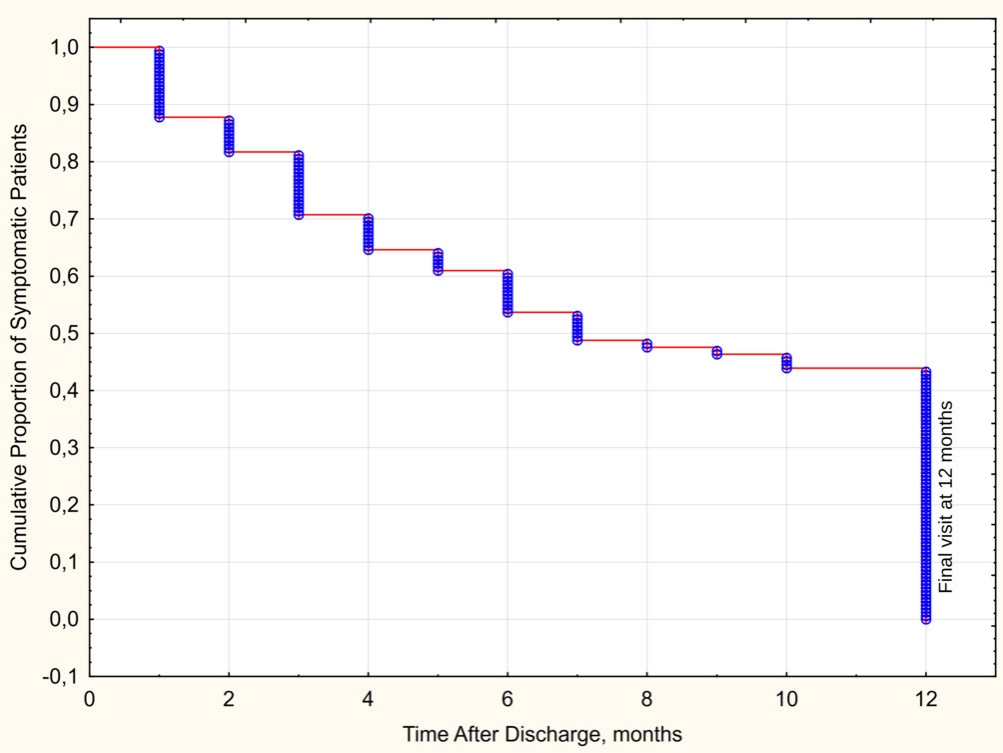
Dynamics of self-reported persistence of symptoms following hospitalization for COVID-19.

Baseline characteristics of the final study cohort participants are presented in Table 1. The vast majority of observed patients remained symptomatic at one month (88%) and three months after discharge (76%), with the rate decreasing to 43% at one year (see Figure 2 for Kaplan–Meier curve of self-reported persistence of symptoms). Patients with ongoing symptoms at 12 months did not differ from their symptom-free counterparts by age, sex, BMI, and major comorbidities (with hypertension and obesity being the most frequent ones) but had a higher cumulative burden of comorbidities as assessed by Charlson index (0.7 ± 1.0 vs. 0.3 ± 0.5, *p* < 0.001), primarily due to the prevalence of less frequent conditions. Both groups were comparable by the extent of radiographic pulmonary involvement, the degree of peripheral oxygen desaturation, and received similar treatment. At the same time, patients with LCS at 12 months were characterized by higher peak in-hospital values of ESR, liver enzymes, and serum creatinine – refer to (9) and Supplementary Table S1 for detailed inter-group comparisons.

**Table 1:** Clinical characteristics of the final study cohort.

Parameter	Value
Subjects, *n*	166
Time from symptoms onset, days	22.6 ± 7.2
Age, years	53.7 ± 13.8
Sex, *n* (%)	
Female	90 (54)
Male	76 (46)
Body mass index, kg/m^2^	29.0 ± 5.2
Active smoking status pre-disease, *n* (%)	23 (14)
Comorbidities, *n* (%)	
Hypertension	65 (39)
Obesity	61 (37)
Type 2 diabetes mellitus	17 (10)
Chronic kidney disease	4 (2)
Bronchial asthma	4 (2)
Chronic obstructive pulmonary disease	3 (2)
Angina pectoris	3 (2)
Chronic liver disease	2 (1)
History of peptic ulcer	12 (7)
History of cancer	7 (4)
History of stroke/transient ischemic attack	6 (4)
Minimal SpO2 during acute COVID-19, %	89 [85; 94]
Pulmonary tissue involvement by CT^a^, %	32.2 ± 20.5
Laboratory parameters	
Peak IL-6, pg/ml	9.8 [3.1; 30.5]
Peak CRP, mg/L	24 [6; 60]
Peak ESR, mm/h	30.8 ± 12.6
Hemoglobin, g/dl	13.9 ± 1.6
Lowest eGFR, ml/min/1.73 m^2^	65.1 ± 18.1
Peak ALT, IU/L	43 [26; 78]
Oxygen supplementation, *n* (%)	
Via nasal cannula	93 (56)
Noninvasive/invasive ventilation	9 (5)
Treatment, *n* (%)	
Dexamethasone	147 (89)
Methylprednisolone pulse therapy	111 (67)
Remdesivir	74 (45)

SpO2, peripheral capillary oxygen saturation; IL-6, interleukin 6; CRP, C-reactive protein; ESR, erythrocyte sedimentation rate.

^a^ As assessed by the simplified RALE score (24).

### 3.2 12-month dynamics of the assessed scales' scores

**Figure 3:**
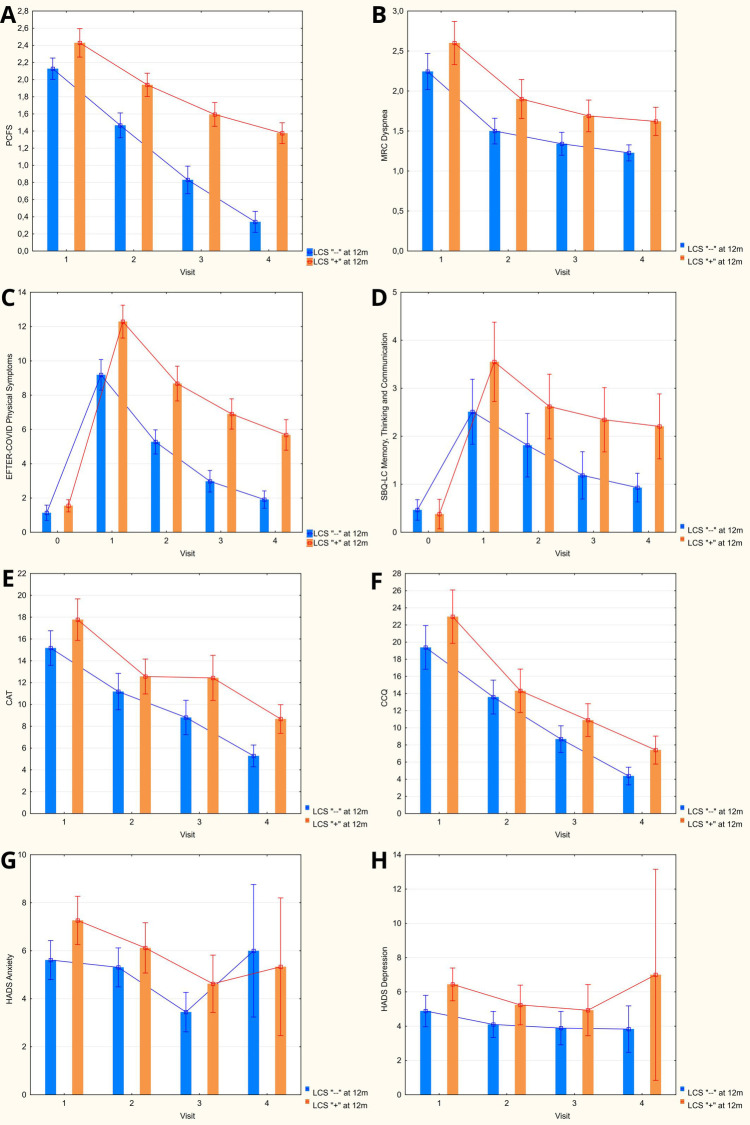
Dynamics of obtained scores on evaluated symptoms scales during 12-months follow-up after hospitalization for COVID-19 (columns: means; whiskers: 95% confidence intervals). **(A)** Post-COVID-19 Functional Status scale. **(B)** Medical Research Council dyspnea scale. **(C)** EFTER-COVID study physical symptoms subscale. **(D)** SBQ-LC Memory, Thinking and Communication subscale. **(E)** COPD assessment test. **(F)** Clinical COPD questionnaire. **(G,H)** Hospital Anxiety and Depression Scale.

Dynamics of the mean scores on evaluated scales at the baseline and throughout one year of follow-up are presented at Figure 3. Among the physical functional scales that were used, PCFS was characterised by a more distinct differentiation between patients with and without LCS symptoms at 1 year, with marginal differences at the baseline getting progrediently larger with each following visit. Despite consistently higher mean scores on MRC Dyspnea scale in the LCS-positive group, the observed differences were only getting marginally significant at 1 and 3 months.

Cumulative physical symptoms score as assessed by respective EFTER-COVID questionnaire subscale was characterized by the most significant inter-group difference among the analyzed indices that was already present at the baseline and persisted throughout the follow-up period. Similar dynamics of the mean values were observed for SBQ-LC Memory, thinking and communication subscale, with higher variability of individual scores contributing to the fact that a statistically significant difference was only detected after 3 months. Despite the continuous trend to normalization of scores on both scales, the mean values at 12 months remained higher compared to the pre-COVID state in both LCS-positive and LCS-negative groups.

Similar to the indices listed above, mean scores on CAT and CCQ, the dedicated respiratory symptoms scales that were used in our study, were characterized by steady progredient improvement between consecutive visits. Out of the two, CAT was showing somewhat better discriminatory capacity, with the borderline differences detected already at 3 months, whereas for CCQ the observed scores in the LCS-positive group were only higher at the final visit.

Both Anxiety and Depression HADS subscales scores were higher in the LCS group during the first visit but similar at every subsequent time points between the patients who completely recovered and those who remained symptomatic at 1 year.

### 3.3 Impact of age on the assessed symptoms scales' scores

**Figure 4:**
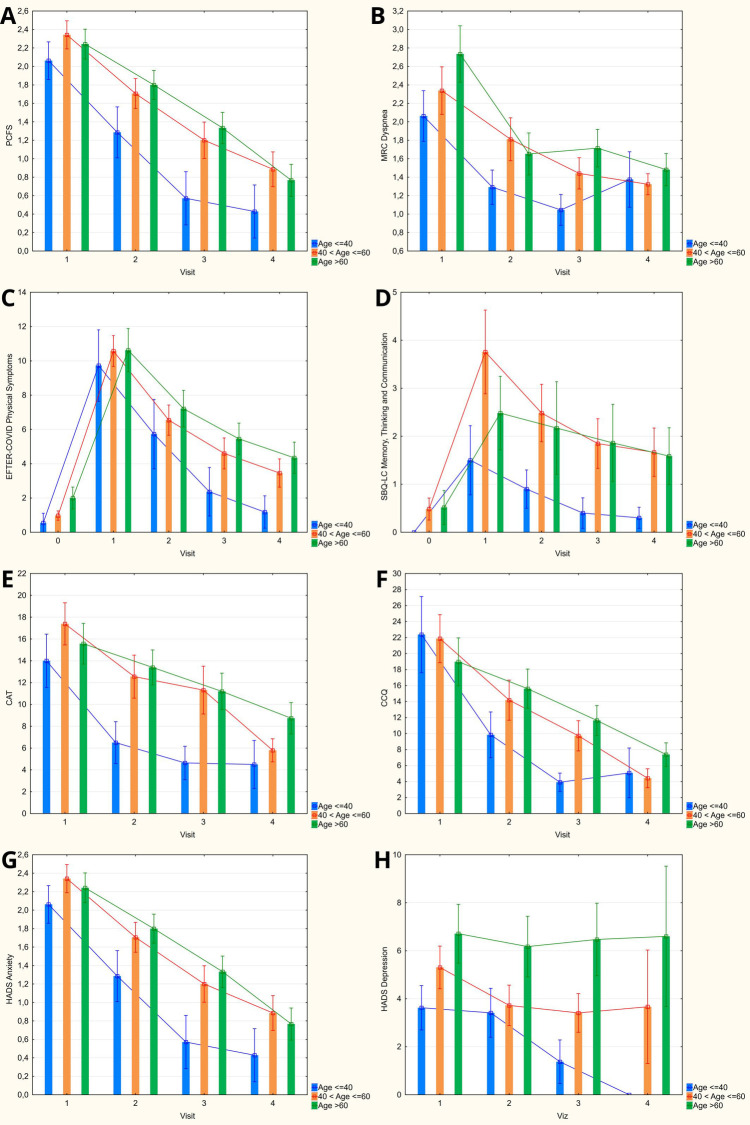
Age categorization of obtained scores on evaluated symptoms scales during 12-months follow-up (columns: means; whiskers: 95% confidence intervals). **(A)** Post-COVID-19 Functional Status scale. **(B)** Medical Research Council dyspnea scale. **(C)** EFTER-COVID study physical symptoms subscale. **(D)** SBQ-LC Memory, Thinking and Communication subscale. **(E)** COPD assessment test. **(F)** Clinical COPD questionnaire. **(G,H)** Hospital Anxiety and Depression Scale.

Categorization of the observed cohort of COVID-19 convalescents by age has, in general, revealed a steeper decrease of mean scores on most assessed scales among younger patients (age up to 40 years) – see Figure 4. This tendency was manifested in lower PCFS, MRC Dyspnea, CAT, and HADS Anxiety scores that were revealed as early as 1 month after discharge, as well as lower baseline SBQ-LC Memory, Thinking and Communication score that persisted at each follow-up visit. EFTER-COVID study physical symptoms subscale and CCQ were characterized by somewhat lesser inter-group differences in dynamics of recovery, with lower mean values for younger patients detected starting from 3 months after discharge. By 12 months, the “delayed” recovery in both older groups brought the scores on respiratory symptoms scales (CAT and CCQ) down to levels comparable to the patients ≤ 40 years old, with the values in the elderly (>60 years old) being significantly higher vs. those aged 41–60. MRC Dyspnea scores at 1 year were also comparable between different age strata. PCFS and both cumulative symptom scores that were used in our study (EFTER-COVID and SBQ-LC) remained lower in younger patients, coming closer to pre-COVID levels, whereas the scores in both older groups were comparable and stayed elevated vs. pre-disease values.

### 3.4 Prediction of long COVID-19 syndrome persistence at 12 months

**Figure 5:**
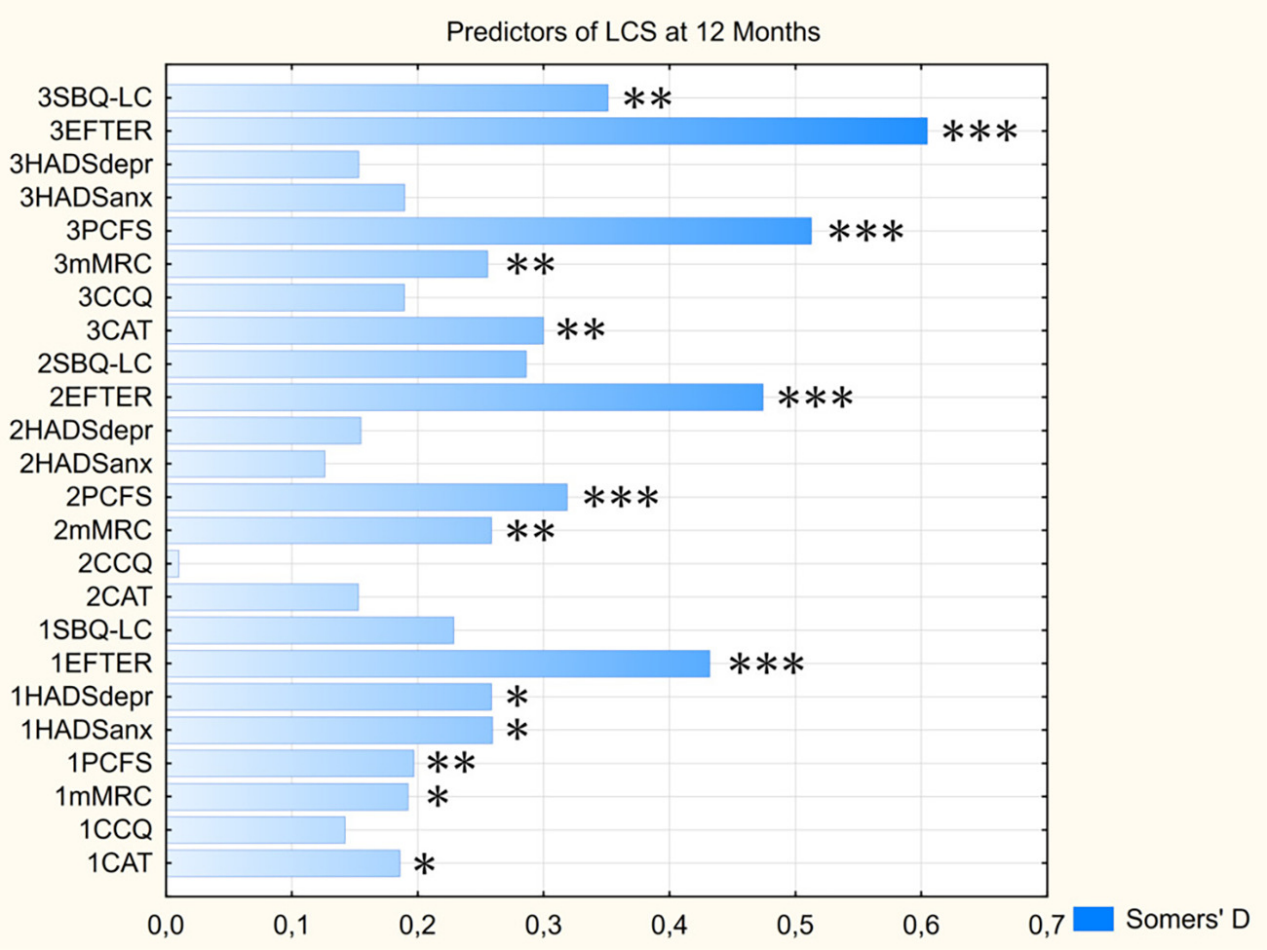
Predictive value of assessed scores for persistence of LCS symptoms at 12 months. Parameters’ prefixes indicate the visit No. CAT, COPD assessment test; CCQ, Clinical COPD questionnaire; EFTER, EFTER-COVID study physical symptoms subscale; HADSanx, Anxiety subscale of Hospital Anxiety and Depression Scale; HADSdepr, Depression subscale of Hospital Anxiety and Depression Scale; MRC, Medical Research Council dyspnea scale; PCFS, Post-COVID-19 Functional Status scale; SBQ-LC, Symptoms Burden Questionnaire – Long COVID Memory, Thinking and Communication subscale. * - *p* < 0.05, ** - *p* < 0.01, *** - *p* < 0.001.

Marginal logistic regression analysis of the scores on assessed symptoms scales that were obtained at different moments of time (see Figure 5 and Supplementary Table S2) has revealed that EFTER-COVID Physical symptoms scale was characterized by the highest and most significant predictive value for the long-term persistence of LCS symptoms at every visit, typically followed by PCFS and MRS Dyspnea scores. For most parameters, their predictive value was increasing with each next visit, with the exceptions being CAT and CCQ with a “gap” at one month post-discharge, and HADS subscales that only were significant predictors at the baseline. Despite the age-related trends described above, age was not predictive of the self-assessed “very long” COVID, nor were the other baseline data including sex, anthropometrical indices, and presence of specific comorbidities (Somers’ *D* < 0.1/*p* > 0.05 for all mentioned parameters).

Employment of machine learning-based classification methods to develop binary predictive models did not result in significant improvement of parameters of the earlier proposed model (9) when limiting the scope of inputs by the time of the first two visits.

Considering the absence of inconsistencies in self-assessed LCS persistence at 3 and 12 months (i.e., no patients who reported complete recovery during Visit 3 had later identified themselves as symptomatic during the final visit), the algorithm to predict the outcome at one year using the inputs obtained at 3 months included the LCS status at Visit 3, yielding a 100% accuracy within the subgroup of patients who were already reporting complete recovery.

**Figure 6:**
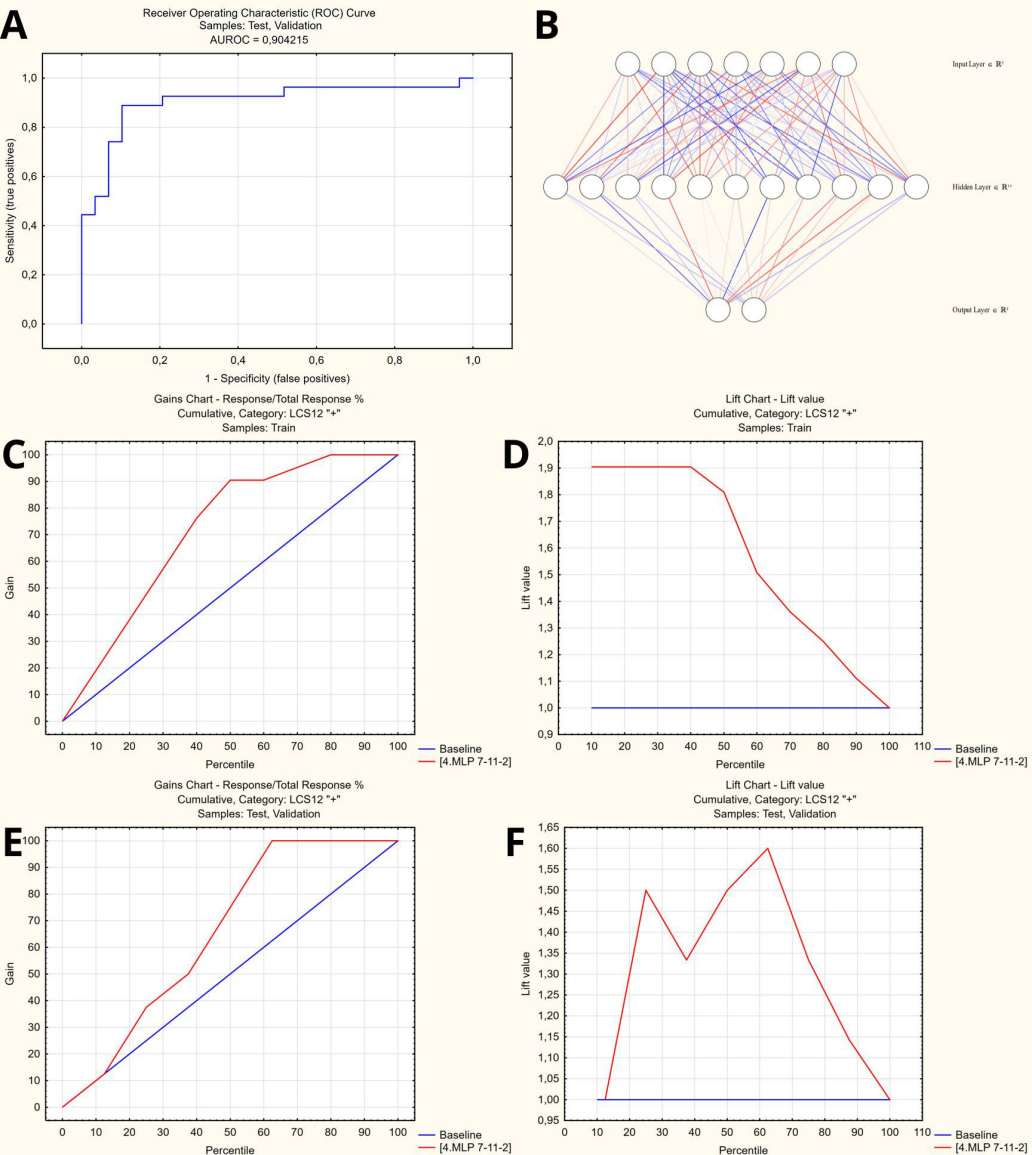
Artificial neural network to predict persisting long COVID symptoms at 12 months. **(A)** Receiver operator characteristic curve (AUROC = 0.904). **(B)** Artificial neural network architecture (SANN 7-11-2). **(C,D)** Gains and lift charts – Training sample. **(E,F)** Gains and lift charts – Test/Validation samples.

For the patients who remained symptomatic, the optimal set of predictors included age, EFTER-COVID Physical symptoms, SBQ-LC Memory, Thinking and Communication, MRC Dyspnea, and CAT scores. The comparative summary of predictive performance of the resulting binary classification models using various machine learning methods is presented at Table 2. The optimal model that outperformed alternative approaches was utilizing SANN 7-11-2 architecture and is available in open access at https://doi.org/10.5281/zenodo.14703690. It yielded a 88% accuracy in the test/validation subset of the study cases, translating (given 24% of completely recovered patients at 3 months) into the 91% overall predictive accuracy in the final study cohort – see Figure 6 for the model characteristics, and supplementary material for instructions and detailed model architecture (refer to Supplementary Table S3 for network weights and connections).

**Table 2:** Comparison of prognostic performance of the optimal model to predict long COVID at 12 months vs. alternative machine learning methods and a SANN model based on the survey at 1 month.

Index	Accuracy, %	Sensitivity, %	Specificity, %	PPV, %	NPV, %
SANN (3 month)	87, 5	94, 1	80, 0	84,2	92, 3
Alternative machine learning methods-based models					
Support vector machine	80, 0	88, 9	70, 6	76, 2	85, 6
Naïve Bayes classifier	71, 4	66, 7	76, 5	75, 0	68, 4
K-nearest neighbors	82, 3	77, 8	87, 5	87, 5	77, 8
Random forest	74, 2	72, 2	76, 9	81, 3	66, 7
Boosted trees	83, 3	77, 8	88, 9	87, 5	80, 0
Neural network-based model using the results of survey obtained at 1 month					
SANN (1 month)	80, 0	87, 5	77, 8	63, 6	93, 3

PPV, positive predictive value; NPV, negative predictive value; SANN, simple artificial neural network.

## 4 Discussion

Long COVID syndrome, which has become an emerging global health concern following the novel SARS-CoV-2 pandemic (1), presents a multifaceted problem. Firstly, the wide range of persistent symptoms experienced by patients who develop LCS, such as chronic fatigue, shortness of breath, cognitive dysfunction, and mental health disorders, can significantly impact their quality of life and ability to return to pre-COVID functioning (2, 19). The economic implications of long COVID should also not be ignored, as it may lead to substantial productivity losses and long-term disability in affected individuals, further adding to the overall societal impact (19, 20). Moreover, the prolonged course of LCS poses a substantial burden on healthcare systems by putting extra strain on the available resources.

Under these conditions, the ability to predict the development of LCS has the potential to improve patient care and healthcare planning. Early identification of individuals at higher risk for developing LCS could enable timely intervention strategies, ensuring prompt initiation of targeted rehabilitation programs, individual support, and emerging dedicated therapies. This forecasting capability could potentially allow healthcare providers to more effectively allocate available resources by taking into account the individualized risk profiles while tailoring care plans for post-acute COVID-19 patients (21, 22).

The current study reports on the results of one-year follow-up of hospitalized COVID-19 survivors that included comprehensive survey-based evaluation pre-discharge, at 1 and 3 months, followed by assessment of a self-reported recovery to pre-COVID state that was performed at 12 months after discharge. The dynamics of symptom scales' scores showed a clear trajectory of improvement over time, with significant differences between patients who reported recovery and those with LCS. For physical symptoms, EFTER-COVID scores in the LCS cohort remained elevated compared to recovered participants, showing steady but incomplete normalization by 12 months. Similarly, univariate scales of physical well-being such as PCFS and, to a lesser extent, MRC Dyspnea, have shown a discriminatory ability between the LCS and LCS-free cohorts throughout the follow-up. SBQ-LC Memory, Thinking, and Communication subscale revealed higher variability but followed a comparable trend, with significant intergroup differences becoming more evident at three months. Out of the two dedicated respiratory symptoms scales used, CAT has demonstrated a better discriminatory ability in our cohort. Emotional status as assessed by HADS subscales was not predictive of the outcome at 12 months. These observations align with the variability of LCS predictors depending on the healthcare setting and terms of assessment, as well as disparities in symptom persistence over extended follow-ups that were reported before (4, 21, 22).

Age-related differences in recovery trajectories were particularly notable. Younger patients (<40 years) exhibited faster and more pronounced improvements in physical and respiratory symptoms, as reflected in Post-COVID Functional Status (PCFS), MRC Dyspnea, CAT, and CCQ scores. These differences were detectable as early as one month post-discharge and remained significant at three months. In contrast, older patients (>60 years) experienced slower recovery, with scores for respiratory and cognitive symptoms staying elevated at one year. These findings corroborate the observations of age being a possible determinant of symptom resolution (21–23), though we did not identify a linear age effect on all scales.

Our results also highlight the importance of survey-based symptom assessments conducted three months after hospital discharge as a pragmatic and effective alternative to pre-discharge evaluations for predicting LCS persistence at 12 months. By leveraging patient-reported outcomes, particularly cumulative physical symptoms scores and scales such as EFTER-COVID and SBQ-LC, the study demonstrated their high predictive value, with EFTER-COVID scores consistently outperforming other measures across all time points. These findings align with data from several studies (13, 15, 17), which emphasized the predictive utility of symptom burden questionnaires in long COVID evaluations.

Compared to pre-discharge assessments which included detailed laboratory and sonographic findings (9), the three-month survey-based evaluation provided simpler yet comparable prognostic insights. Incorporating the assessment of a self-reported recovery status at 3 months followed by employing the ML-based classification model that utilizes age, EFTER-COVID Physical symptoms, SBQ-LC Memory, Thinking and Communication, MRC Dyspnea, and CAT scores as predictors of persistence of symptoms at 1 year has allowed to achieve a 91% predictive accuracy in the validation cohort. Compared to the similarly performing pre-discharge model (9) that was presented previously and required, along with routinely available laboratory parameters, several tests beyond the standard of care for acute COVID-19, the approach presented here alleviates the possible problem with logistics of auxiliary assessment by postponing it and transferring to the primary care setting.

In conclusion, the study underscores the potential of survey-based symptom assessment at three months post-discharge as an accessible, reliable, and cost-effective method for identifying individuals at risk for prolonged LCS. This scalable approach not only simplifies long-term management but also supports timely and targeted rehabilitation efforts across diverse healthcare settings.

### 4.1 Study limitations

This study has several limitations that should be considered when interpreting its findings. First, it was conducted at a single specialized COVID-19 care center, which may introduce center-specific effects and, together with a smaller sample size, may limit the generalizability of the results. While the observed outcomes were not significantly influenced by variations in treatment, such as the use of methylprednisolone pulse therapy, the findings should be validated in diverse healthcare settings. Second, the exclusion of individuals with severe underlying pathologies was aimed at minimizing confounding effects but may have introduced selection bias, potentially underestimating the true burden of long COVID syndrome. However, the prevalence of major comorbidities in the study cohort aligns with existing literature, suggesting the representativeness of the sample.

Another limitation stems from the evolving SARS-CoV-2 landscape. Changes in viral variants and the increasing prevalence of vaccination since the study period may affect the applicability of these results to current clinical scenarios. These shifts necessitate caution in extrapolating the findings to broader populations. Future research should address these limitations by including more diverse cohorts, accounting for variations in treatment protocols, and exploring the impact of emerging viral variants and vaccination on long COVID trajectories.

## 5 Conclusions

This study demonstrates that survey-based symptom assessments conducted at three months post-discharge are a robust and practical alternative to complex pre-discharge evaluations for predicting the persistence of long COVID syndrome (LCS) at 12 months. EFTER-COVID Study Physical Symptoms and SBQ-LC Memory, Thinking, and Communication subscales and PCFS class at 3 months emerged as the most reliable predictors, effectively distinguishing between patients who had subsequently recovered and those with LCS at 1 year. Age-related differences revealed faster recovery in younger patients, while older individuals exhibited prolonged respiratory and cognitive symptoms. A machine learning model incorporating survey-based scores at three months achieved 91% predictive accuracy which was comparable to pre-discharge models but associated with significantly reduced logistical complexity. These findings highlight the potential of accessible, patient-centered evaluations in guiding long-term care and rehabilitation strategies for COVID-19 survivors, particularly in resource-limited settings.

## Data Availability

The raw data supporting the conclusions of this article will be made available by the authors, without undue reservation.
