# Supplementary material for: 12-Month trajectories of physical and mental symptom scores after COVID-19 hospitalization and their role in predicting “very long” COVID
Source: Front Rehabil Sci. 2025 May 21;6:1568291. doi: 10.3389/fresc.2025.1568291 (PMC12133859; doi:10.3389/fresc.2025.1568291)
Supplement: Supplementary file 1 [file Datasheet1.pdf]

Supplementary Material

12-Month Trajectories of Physical and Mental Symptom Scores After COVID-19 Hospitalization and Their Role in Predicting "Very Long" COVID

Oleksii Honchar*, Tetiana Ashcheulova, Alla Bobeiko, Viktor Blazhko, Eduard Khodosh, Nataliia Matiash, Vladyslav Syrota

*** Correspondence:**
Oleksii Honchar at ov.honchar@knmu.edu.ua

2 Material and Methods

Study exclusion criteria:

- stage D chronic heart failure;
- acute heart failure;
- history of myocardial infarction;
- permanent atrial fibrillation;
- stroke within 6 months;
- severe uncontrolled hypertension;
- significant valvular heart disease;
- active cancer or systemic autoimmune pathology;
- inability to provide informed consent;
- persisting O2 supplementation dependence by the time of discharge.

3 Results

The source file of the machine learning based binary classification model is available in open access at https://doi.org/10.5281/zenodo.14703690 and requires an input dataset with the following variables:

- Age = age at the time of disease onset, years;
- 3CAT = COPD assessment test score at 3 months after hospital discharge;
- 3EFTER-COVID Physical = EFTER-COVID study subscale on physical symptoms score at 3 months;
- 3SBQ-LC = Symptoms Burden Questionnaire – Long COVID Memory, Thinking and Communication subscale score at 3 months after hospital discharge;
- 3MRC = Medical Research Council Dyspnea scale self-assessment at 3 months.

Output codes include "No" for absence and "Yes" for the presence of long COVID-19 symptoms at 12 months among patients who remained symptomatic at 3 months after hospital discharge.

Supplementary Tables and Figures

Supplementary table 1. Demographics and pre-discharge clinical characteristics of the study participants with and without persisting long COVID syndrome at 12 months.

Parameters	No long COVID at 12 months	Long COVID at 12 months	Difference (95% CI)	2-sided p
Subjects, n (%)	95 (57)	71 (43)		
Female sex, n (%)	51 (54)	39 (55)		0,873
Age, years	52,9 ± 14,5	54,7 ± 12,8	1,8 (-2,5; 6,1)	0,409
Long COVID at 3 months, n (%)	55 (58)	71 (100)		< 0,001
BMI, kg/m^2	28,8 ± 5,3	29,3 ± 5,0	0,5 (-1,1; 2,1)	0,529
Comorbidities, n (%)				
Hypertension	38 (40)	27 (38)		0,797
Obesity	34 (36)	27 (38)		0,767
Diabetes mellitus, type 2	9 (9)	8 (11)		0,706
History of peptic ulcer	4 (4)	8 (11)		0,082
History of cancer	2 (2)	5 (7)		0,117
History of stroke / TIA	3 (3)	3 (4)		0,715
Chronic kidney disease	0 (0)	4 (6)		0,019
Bronchial asthma	0 (0)	4 (6)		0,019
COPD	3 (3)	0 (0)		0,131
Angina pectoris	0 (0)	3 (4)		0,043
Chronic liver disease	0 (0)	2 (3)		0,100
Charlson comorbidity index	0,31 ± 0,51	0,69 ± 0,96	0,37 (0,15; 0,60)	0,001
Active smoking status, n (%)	17 (18)	6 (8)		0,081
Hospitalization period, days	14,6 ± 4,1	15,8 ± 5,1		0,106
Pulmonary involvement by CT, %	34,0 ± 21,9	30,1 ±18,8	-3,9 (-12,1; 4,3)	0,344
Minimal in-hospital SpO2, %	88,7 ± 7,5	87,4 ± 7,8	-1,3 (-3,7; 1,1)	0,277
Oxygen supplementation, n (%)				
Via nasal cannula	48 (51)	45 (63)		0,099
Assisted ventilation	6 (6)	3 (4)		0,556
Laboratory parameters				
Peak IL-6, pg/mL	10,0 [3,2; 25,2]	9,7 [3,1; 47,0]		0,843
Peak CRP, mg/L	16 [6; 48]	33 [7; 67]		0,412
Peak ESR, mm/h	28,3 ± 12,6	34,3 ± 12,1	6,1 (1,6; 10,5)	0,008
Peak procalcitonin, ng/mL	0,06 [0,05; 0,08]	0,05 [0,03; 0,12]		0,521
Peak D-dimer, ng/mL	287 [199; 494]	179 [128; 628]		0,197
Peak ALT, IU/L	46,3 ± 25,3	76,3 ± 60,8	30,0 (11,0; 49,0)	0,002
Peak AST, IU/L	33,0 ± 17,6	52,5 ± 38,9	19,5 (7,2; 31,9)	0,002
Peak creatinine, μmol/L	97,4 ± 17,0	109,7 ± 26,2	12,3 (3,5; 21,2)	0,007
Lowest eGFR, $ml/min/1{,}73m^2$	69,8 ± 20,7	59,5 ± 14,7	-10,3 (-17,7; -2,9)	0,007
Hemoglobin, g/dL	13,9 ± 1,7	13,9 ± 1,3	-3,1 (-9,4; 3,3)	0,760
Treatment, n (%)				
Dexamethasone	85 (89)	62 (87)		0,667
Methylprednisolone	64 (67)	47 (66)		0,874
Remdesivir	42 (44)	32 (45)		0,912

Values presented as Mean ± SD or Median (IQR). BMI – body mass index, TIA – transient ischemic attack, COPD – chronic obstructive pulmonary disease, CT – computed tomography, SpO2 – peripheral capillary oxygen saturation, IL-6 – interleukin 6, CRP – C-reactive protein, ESR – erythrocyte sedimentation rate, ALT – alanine aminotransferase, eGFR – estimated glomerular filtration rate by CKD-EPI equation.

Supplementary table 2. Marginal analysis of potential survey-based predictors of persisting Long COVID-19 symptoms at 12 months in logistic regression analysis.

Parameters	Long COVID at 12 months - Marginal table Analysis using full sample, n=166			
	Somers' D	Estimate	Pr>Chi.Sqr	df
CAT, visit 1	0,18595	0,045298008	0,0392652569	1
CCQ, visit 1	0,142222	0,0226980103	0,0760548584	1
MRC, visit 1	0,192381	0,291673057	0,0474224226	1
PCFS, visit 1	0,19696	0,72383781	0,0049430313	1
HADSanx, visit 1	0,259358	0,104179196	0,0130837061	1
HADSdepr, visit 1	0,25869	0,0889678427	0,0245316206	1
EFTER-COVID, visit 1	0,431818	0,186207724	0,0000395958701	1
EFTER-COVID at visit 1, question 1	0,083578	-1,13497993	0,0746648539	1
EFTER-COVID at visit 1, question 2	0,153226	0,537044332	0,0957325982	1
EFTER-COVID at visit 1, question 3	0,247801	1,28093385	0,0016799031	1
EFTER-COVID at visit 1, question 4	0,220674	0,933576368	0,00684428189	1
EFTER-COVID at visit 1, question 5	0,280059	1,54181772	0,000411998293	1
EFTER-COVID at visit 1, question 6	0,045455	0,965791198	0,241671828	1
EFTER-COVID at visit 1, question 7	0,190341	0,784408772	0,0197966251	1
EFTER-COVID at visit 1, question 8	0,070381	0,261853547	0,473895611	1
EFTER-COVID at visit 1, question 9	0,302053	1,43953888	0,000126055696	1
EFTER-COVID at visit 1, question 10	0,116477	0,4704614	0,156659209	1
EFTER-COVID at visit 1, question 11	0,019886	0,0831339002	0,804421458	1
EFTER-COVID at visit 1, question 12	0,199413	1,40876722	0,00242368883	1
EFTER-COVID at visit 1, question 13	0,228739	1,04982212	0,00472730171	1
EFTER-COVID at visit 1, question 14	0,052557	0,482273578	0,351706384	1
EFTER-COVID at visit 1, question 15	0,11437	0,521296924	0,140795184	1
EFTER-COVID at visit 1, question 16	0,015396	0,144581229	0,775211533	1
EFTER-COVID at visit 1, question 17	0,16129	0,85393397	0,0411914845	1
EFTER-COVID at visit 1, question 18	0,005865	0,0689928715	0,903232077	1
EFTER-COVID at visit 1, question 19	0,177419	0,741937345	0,0316830623	1
EFTER-COVID at visit 1, question 20	0,196481	0,873731117	0,0154090778	1
EFTER-COVID at visit 1, question 21	0,294721	1,22167238	0,000429377515	1
EFTER-COVID at visit 1, question 22	0,095238	0,470003629	0,210080902	1
EFTER-COVID at visit 1, question 23	0,111111	21,6806552	0,998969166	1
SBQ-LC, visit 1	0,228549	0,103482507	0,0584014422	1
SBQ-LC at visit 1, question 1	0,133921	0,24781613	0,171577628	1
SBQ-LC at visit 1, question 2	0,036889	-0,74104085	0,212251139	1
SBQ-LC at visit 1, question 3	-0,009623	-0,0234540795	0,907051422	1
SBQ-LC at visit 1, question 4	0,057739	0,334131084	0,256333615	1
SBQ-LC at visit 1, question 5	0,102646	0,437545751	0,0773957204	1

SBQ-LC at visit 1, question 6	0,0417	0,15201818	0,675564442	1
SBQ-LC at visit 1, question 7	0,169206	0,495670105	0,0430189835	1
SBQ-LC at visit 1, question 8	0,056937	0,860940637	0,198393074	1
SBQ-LC at visit 1, question 9	0,085806	0,609288368	0,0579151752	1
SBQ-LC at visit 1, question 10	0,046512	1,08222066	0,13761186	1
CAT, visit 2	0,152821	0,0303181527	0,261137675	1
CCQ, visit 2	0,010256	0,00944924884	0,646173501	1
MRC, visit 2	0,258462	0,642734971	0,00618748227	1
PCFS, visit 2	0,31875	1,25729642	0,000115640085	1
HADSanx, visit 2	0,126154	0,062020001	0,218277429	1
HADSdepr, visit 2	0,154872	0,0838582957	0,0909989249	1
EFTER-COVID, visit 2	0,47434	0,255632539	0,00000234285736	1
EFTER-COVID at visit 2, question 1	0,074047	1,52760789	0,0671184157	1
EFTER-COVID at visit 2, question 2	0,138563	2,11254149	0,00782009615	1
EFTER-COVID at visit 2, question 3	0,110704	0,465089614	0,171526639	1
EFTER-COVID at visit 2, question 4	0,292522	1,47085175	0,00014654011	1
EFTER-COVID at visit 2, question 5	0,32478	1,26507309	0,000310873544	1
EFTER-COVID at visit 2, question 6	0,159091	1,93620897	0,0109072123	1
EFTER-COVID at visit 2, question 7	0,258523	1,1059924	0,001411759	1
EFTER-COVID at visit 2, question 8	0,021261	0,0136396926	0,973633528	1
EFTER-COVID at visit 2, question 9	0,199413	1,40876722	0,00242368883	1
EFTER-COVID at visit 2, question 10	0,133523	0,601339631	0,0852247427	1
EFTER-COVID at visit 2, question 11	0,113636	0,747214402	0,0780591356	1
EFTER-COVID at visit 2, question 12	0,074047	1,52760789	0,0671184157	1
EFTER-COVID at visit 2, question 13	0,360704	2,10842908	0,00000281411907	1
EFTER-COVID at visit 2, question 14	0,09375	0,587786665	0,164891212	1
EFTER-COVID at visit 2, question 15	0,195748	1,06717076	0,00678625681	1
EFTER-COVID at visit 2, question 16	0,009531	0,360002734	0,722578156	1
EFTER-COVID at visit 2, question 17	0,115836	1,39586381	0,023758365	1
EFTER-COVID at visit 2, question 18	0,028592	0,381367557	0,526965676	1
EFTER-COVID at visit 2, question 19	0,134897	1,07044141	0,0255370221	1
EFTER-COVID at visit 2, question 20	0,277859	1,16825472	0,000934155881	1
EFTER-COVID at visit 2, question 21	0,262463	1,08756245	0,001590037	1
EFTER-COVID at visit 2, question 22	0,142857	0,753771802	0,0533501466	1
EFTER-COVID at visit 2, question 23	0,045045	-0,196710294	0,600098156	1
SBQ-LC, visit 2	0,286287	0,0967529489	0,112468008	1
SBQ-LC at visit 2, question 1	0,219727	0,405943596	0,0349188617	1
SBQ-LC at visit 2, question 2	0,011227	0,405465108	0,689478541	1
SBQ-LC at visit 2, question 3	-0,003208	-0,0915122484	0,777007481	1
SBQ-LC at visit 2, question 4	-0,019246	-0,189463085	0,697960772	1
SBQ-LC at visit 2, question 5	0,117081	0,569675896	0,0348797352	1
SBQ-LC at visit 2, question 6	0,011227	0,129422976	0,754073319	1

Supplementary table 2 (continued).

SBQ-LC at visit 2, question 7	0,189254	0,57823183	0,0407561638	1
SBQ-LC at visit 2, question 8	0,077787	0,608760583	0,28755532	1
SBQ-LC at visit 2, question 9	0,059342	-1,01757466	0,164625134	1
SBQ-LC at visit 2, question 10	0,04571	1,13497993	0,19887506	1
CAT, visit 3	0,3	0,0607664315	0,0073764851	1
CCQ, visit 3	0,189394	0,0404824718	0,0760277223	1
MRC, visit 3	0,255486	0,670669795	0,00549411209	1
PCFS, visit 3	0,512633	1,48266056	0,0000000554917279	1
HADSanx, visit 3	0,189815	0,120069007	0,0959851673	1
HADSdepr, visit 3	0,153086	0,0743402537	0,221808716	1
EFTER-COVID, visit 3	0,604839	0,350676571	0,0000000122847109	1
EFTER-COVID at visit 3, question 1	0	0		1
EFTER-COVID at visit 3, question 2	0,129032	21,7474818	0,998812776	1
EFTER-COVID at visit 3, question 3	0,228006	1,20547543	0,00205188062	1
EFTER-COVID at visit 3, question 4	0,27346	1,5945914	0,00017674099	1
EFTER-COVID at visit 3, question 5	0,352639	1,31476574	0,000117070839	1
EFTER-COVID at visit 3, question 6	0,448864	2,02595286	0,000000197061689	1
EFTER-COVID at visit 3, question 7	0,332386	1,72066355	0,0000161812547	1
EFTER-COVID at visit 3, question 8	0,010264	-0,202541464	0,642548812	1
EFTER-COVID at visit 3, question 9	0,189883	1,5589071	0,00237208573	1
EFTER-COVID at visit 3, question 10	0,122159	0,726738125	0,0711878388	1
EFTER-COVID at visit 3, question 11	0,119318	1,14862271	0,0303733818	1
EFTER-COVID at visit 3, question 12	0,064516	21,6760229	0,999163264	1
EFTER-COVID at visit 3, question 13	0,267595	2,86738224	0,000188990169	1
EFTER-COVID at visit 3, question 14	0,360795	1,51566501	0,0000165689807	1
EFTER-COVID at visit 3, question 15	0,222141	1,7211419	0,000688480106	1
EFTER-COVID at visit 3, question 16	0	0		1
EFTER-COVID at visit 3, question 17	0,106305	1,85165761	0,0221573233	1
EFTER-COVID at visit 3, question 18	0,019062	0,370373788	0,610641599	1
EFTER-COVID at visit 3, question 19	0,022727	-19,9201361	0,999098239	1
EFTER-COVID at visit 3, question 20	0,343842	1,5522795	0,000023864268	1
EFTER-COVID at visit 3, question 21	0,307918	1,30625165	0,00020196904	1
EFTER-COVID at visit 3, question 22	0,166667	0,916290732	0,0222758751	1
EFTER-COVID at visit 3, question 23	0,102102	-0,518793793	0,206701071	1
SBQ-LC, visit 3	0,351243	0,205135306	0,00963888612	1
SBQ-LC at visit 3, question 1	0,249399	0,469420775	0,017291667	1
SBQ-LC at visit 3, question 2	-0,010425	-0,184742256	0,786499443	1
SBQ-LC at visit 3, question 3	0,0417	0,15201818	0,675564442	1
SBQ-LC at visit 3, question 4	0,011227	0,405465108	0,689478541	1
SBQ-LC at visit 3, question 5	0,185245	0,986445446	0,00423108517	1

SBQ-LC at visit 3, question 6	0,068966	20,327877	0,998884533	1
SBQ-LC at visit 3, question 7	0,157979	1,11402576	0,00568645345	1
SBQ-LC at visit 3, question 8	0,077787	0,608760583	0,28755532	1
SBQ-LC at visit 3, question 9	0,035285	-0,741937345	0,374177263	1
SBQ-LC at visit 3, question 10	0,04571	1,13497993	0,19887506	1

CAT, COPD assessment test; CCQ, Clinical COPD questionnaire; EFTER, EFTER-COVID study physical symptoms subscale; HADSanx, Anxiety subscale of Hospital Anxiety and Depression Scale; HADSdepr, Depression subscale of Hospital Anxiety and Depression Scale; MRC, Medical Research Council dyspnea scale; PCFS, Post-COVID-19 Functoinal Status scale; SBQ-LC, Symptoms Burden Questionnaire – Long COVID Memory, Thinking and Communication subscale.

Supplementary table 3. Connections and weight values of the machine learning model to predict persisting Long COVID-19 symptoms at 12 months.

Weight ID	Network weights	
	Connections<br>1.MLP 7-11-2	Weight values<br>1.MLP 7-11-2
1	Age --> hidden neuron 1	0,58739
2	3CAT --> hidden neuron 1	0,44675
3	3EFTER-COVID Physical --> hidden neuron 1	0,20976
4	3SBQ-LC --> hidden neuron 1	-0,08377
5	3MRC(1) --> hidden neuron 1	0,17358
6	3MRC(2) --> hidden neuron 1	-0,44133
7	3MRC(3) --> hidden neuron 1	-0,00281
8	Age --> hidden neuron 2	0,36818
9	3CAT --> hidden neuron 2	0,19645
10	3EFTER-COVID Physical --> hidden neuron 2	-0,67129
11	3SBQ-LC --> hidden neuron 2	0,00540
12	3MRC(1) --> hidden neuron 2	-0,16414
13	3MRC(2) --> hidden neuron 2	0,13526
14	3MRC(3) --> hidden neuron 2	0,06342
15	Age --> hidden neuron 3	0,92775
16	3CAT --> hidden neuron 3	1,96869
17	3EFTER-COVID Physical --> hidden neuron 3	0,55855
18	3SBQ-LC --> hidden neuron 3	-0,51957
19	3MRC(1) --> hidden neuron 3	0,01339
20	3MRC(2) --> hidden neuron 3	-0,41944
21	3MRC(3) --> hidden neuron 3	-0,57952
22	Age --> hidden neuron 4	0,22237
23	3CAT --> hidden neuron 4	0,20655
24	3EFTER-COVID Physical --> hidden neuron 4	-0,57596
25	3SBQ-LC --> hidden neuron 4	0,54681
26	3MRC(1) --> hidden neuron 4	0,13166
27	3MRC(2) --> hidden neuron 4	-0,11652
28	3MRC(3) --> hidden neuron 4	0,03745
29	Age --> hidden neuron 5	1,30847
30	3CAT --> hidden neuron 5	2,26011
31	3EFTER-COVID Physical --> hidden neuron 5	-2,03367
32	3SBQ-LC --> hidden neuron 5	1,86785
33	3MRC(1) --> hidden neuron 5	-0,31425
34	3MRC(2) --> hidden neuron 5	-0,18694
35	3MRC(3) --> hidden neuron 5	-0,22970

36	Age --> hidden neuron 6	0,78193
37	3CAT --> hidden neuron 6	0,14197
38	3EFTER-COVID Physical --> hidden neuron 6	-0,76670
39	3SBQ-LC --> hidden neuron 6	0,19627
40	3MRC(1) --> hidden neuron 6	-0,52829
41	3MRC(2) --> hidden neuron 6	0,48500
42	3MRC(3) --> hidden neuron 6	-0,01441
43	Age --> hidden neuron 7	-0,64403
44	3CAT --> hidden neuron 7	0,31440
45	3EFTER-COVID Physical --> hidden neuron 7	-1,89288
46	3SBQ-LC --> hidden neuron 7	1,60274
47	3MRC(1) --> hidden neuron 7	-0,69005
48	3MRC(2) --> hidden neuron 7	0,69128
49	3MRC(3) --> hidden neuron 7	0,42244
50	Age --> hidden neuron 8	-0,33305
51	3CAT --> hidden neuron 8	-0,65523
52	3EFTER-COVID Physical --> hidden neuron 8	1,17405
53	3SBQ-LC --> hidden neuron 8	-0,42237
54	3MRC(1) --> hidden neuron 8	-0,40819
55	3MRC(2) --> hidden neuron 8	0,43550
56	3MRC(3) --> hidden neuron 8	0,28459
57	Age --> hidden neuron 9	0,42718
58	3CAT --> hidden neuron 9	-0,04398
59	3EFTER-COVID Physical --> hidden neuron 9	-0,70439
60	3SBQ-LC --> hidden neuron 9	-0,61214
61	3MRC(1) --> hidden neuron 9	-0,31753
62	3MRC(2) --> hidden neuron 9	0,49946
63	3MRC(3) --> hidden neuron 9	0,16736
64	Age --> hidden neuron 10	-1,60300
65	3CAT --> hidden neuron 10	-1,24862
66	3EFTER-COVID Physical --> hidden neuron 10	-1,19981
67	3SBQ-LC --> hidden neuron 10	-1,40187
68	3MRC(1) --> hidden neuron 10	0,63891
69	3MRC(2) --> hidden neuron 10	-0,38216
70	3MRC(3) --> hidden neuron 10	-0,20053
71	Age --> hidden neuron 11	0,72817
72	3CAT --> hidden neuron 11	0,37820
73	3EFTER-COVID Physical --> hidden neuron 11	0,24718
74	3SBQ-LC --> hidden neuron 11	-0,16432
75	3MRC(1) --> hidden neuron 11	0,05554
76	3MRC(2) --> hidden neuron 11	-0,34847

77	3MRC(3) --> hidden neuron 11	0,07584
78	input bias --> hidden neuron 1	-0,27870
79	input bias --> hidden neuron 2	0,02278
80	input bias --> hidden neuron 3	-0,95555
81	input bias --> hidden neuron 4	0,15769
82	input bias --> hidden neuron 5	-0,67142
83	input bias --> hidden neuron 6	-0,19146
84	input bias --> hidden neuron 7	0,27212
85	input bias --> hidden neuron 8	0,24342
86	input bias --> hidden neuron 9	0,19839
87	input bias --> hidden neuron 10	-0,03775
88	input bias --> hidden neuron 11	-0,29134
89	hidden neuron 1 --> LCS12(No)	-0,10961
90	hidden neuron 2 --> LCS12(No)	0,06785
91	hidden neuron 3 --> LCS12(No)	-0,44133
92	hidden neuron 4 --> LCS12(No)	-0,11353
93	hidden neuron 5 --> LCS12(1No)	0,59714
94	hidden neuron 6 --> LCS12(No)	0,20792
95	hidden neuron 7 --> LCS12(No)	-1,09817
96	hidden neuron 8 --> LCS12(No)	0,16008
97	hidden neuron 9 --> LCS12(No)	0,39042
98	hidden neuron 10 --> LCS12(No)	0,58913
99	hidden neuron 11 --> LCS12(No)	-0,08232
100	hidden neuron 1 --> LCS12(Yes)	0,14350
101	hidden neuron 2 --> LCS12(Yes)	-0,11448
102	hidden neuron 3 --> LCS12(Yes)	0,53721
103	hidden neuron 4 --> LCS12(Yes)	0,21069
104	hidden neuron 5 --> LCS12(Yes)	-0,65887
105	hidden neuron 6 --> LCS12(Yes)	-0,17876
106	hidden neuron 7 --> LCS12(Yes)	1,18328
107	hidden neuron 8 --> LCS12(Yes)	-0,18840
108	hidden neuron 9 --> LCS12(Yes)	-0,45582
109	hidden neuron 10 --> LCS12(Yes)	-0,52326
110	hidden neuron 11 --> LCS12(Yes)	0,07903
111	hidden bias --> LCS12(No)	0,33263
112	hidden bias --> LCS12(Yes)	0,49366

Note. Training algorithm = BFGS 37; Error function = SOS; Hidden activation = Exponential; Output activation = Identity. 3CAT, COPD assessment test score at 3 months; 3EFTER-COVID Physical, EFTER-COVID study subscale on physical symptoms score at 3 months; 3MRC, Medical Research Council Dyspnea scale self-assessment at 3 months; 3SBQ-LC, Symptoms Burden Questionnaire – Long COVID Memory, Thinking and Communication subscale score at 3 months; LCS12, persisting long COVID symptoms at 12 months.
